# Spontaneous Resolution of Diffuse Idiopathic Slit-Like “Syrinx” in a Pediatric Patient

**DOI:** 10.7759/cureus.17808

**Published:** 2021-09-07

**Authors:** Ryan K Rigsby, Karen A Tong

**Affiliations:** 1 Radiology, Loma Linda University Medical Center, Loma Linda, USA

**Keywords:** syrinx, syringomyelia, hydromyelia, slit-like, persistent central canal, spontaneous resolution

## Abstract

It is important to recognize that a prominent central canal of the spinal cord can be a normal variant and can spontaneously regress. A five-year-old male presented for evaluation of abnormal gait. Prior brain magnetic resonance imaging showed no hindbrain malformation, and the patient had no history of trauma. Full spine magnetic resonance imaging showed a vertical slit-like linear cavity within the center of the spinal cord, from C6-7 to the conus medullaris with a diameter ranging from 0.5 to 2 mm. This was initially reported as a syrinx. The patient’s symptoms remained stable. Three years later, follow-up magnetic resonance imaging showed spontaneous resolution of the slit-like cavity. This case likely represented a prominent central canal (a normal variant) that underwent normal closure.

## Introduction

*Hydromyelia* is defined as the abnormal expansion of the central canal of the spinal cord. *Syringomyelia* is spinal cord parenchymal fluid accumulation off midline not lined with ependyma. The terms *syrinx* and *syringohydromyelia* can include either hydromyelia or syringomyelia. Prominent* *central canal is a more general term that does not necessarily imply pathology. Causes of syrinx include posterior fossa abnormalities (most notably Chiari malformations) and trauma, though cases can be idiopathic. While there have been many cases of spontaneous regression of syrinx in the settings of posterior fossa abnormalities and trauma, few cases have been reported when the etiology was unknown. We present a case of a five-year-old male without posterior fossa abnormalities or a history of significant trauma, who had spontaneous resolution of a prominent central canal on a three-year follow-up. History was obtained from chart review. An institutional review board waiver was obtained.

## Case presentation

**Figure 1:**
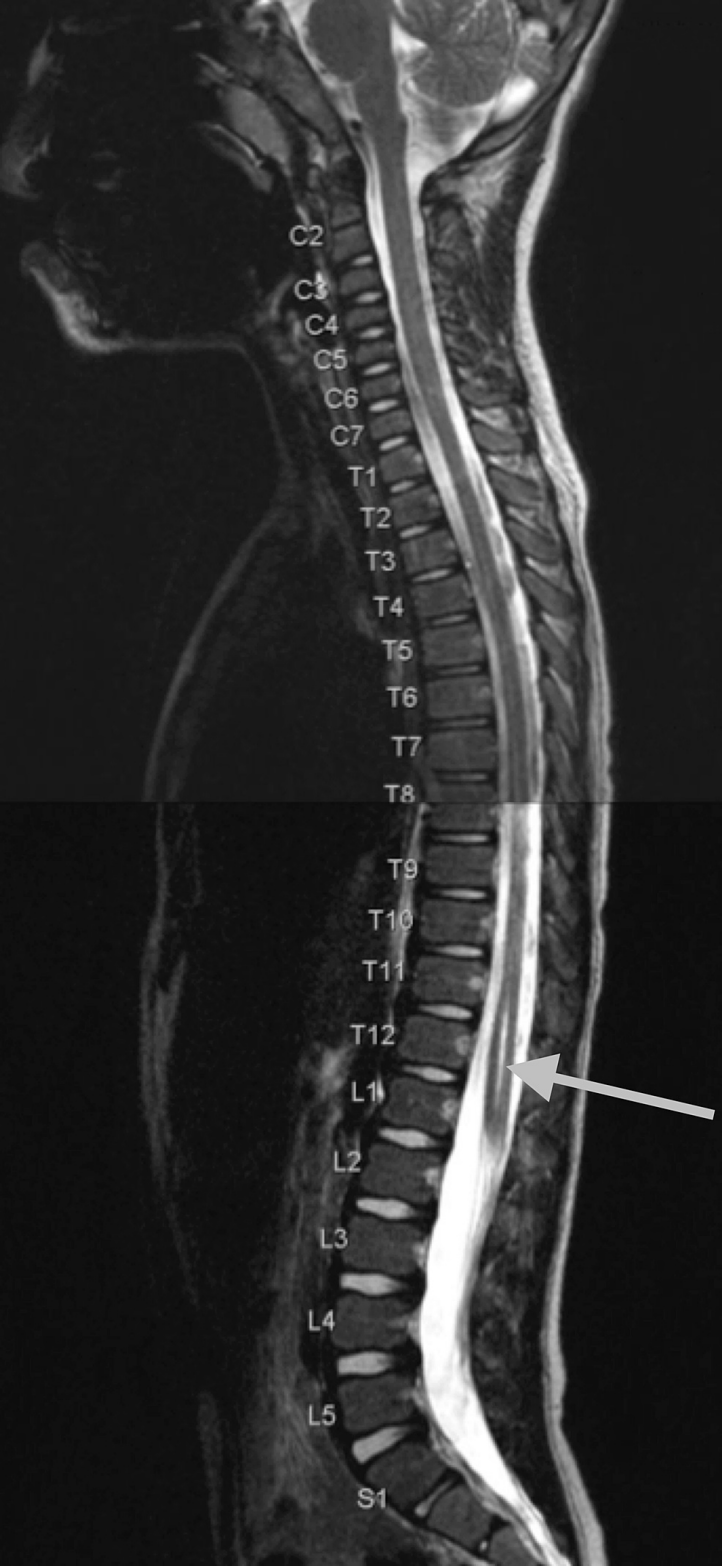
Stitched sagittal T2-weighted images of the cervical, thoracic, and lumbar spine numbered by level at five years of age show a slit-like syrinx from the conus medullaris and tapering cranially to C6-7, which is most prominent in the lower cord (gray arrow).

**Figure 2:**
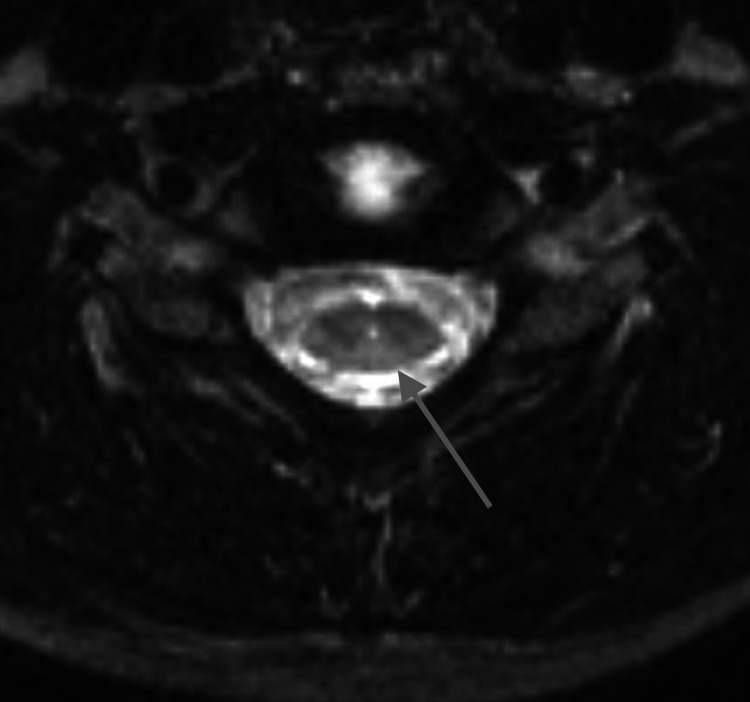
Axial T2-weighted image of the spine at the level of C6-7 at five years of age shows a slit-like syrinx (gray arrow)

**Figure 3:**
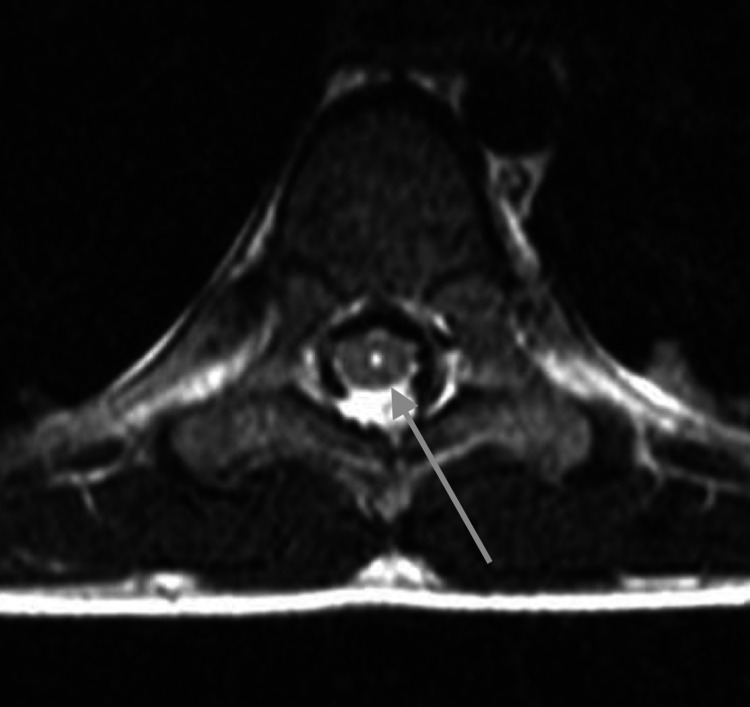
Representative axial T2-weighted image of the spine at the level of T9 at five years of age shows a slit-like syrinx (gray arrow)

**Figure 4:**
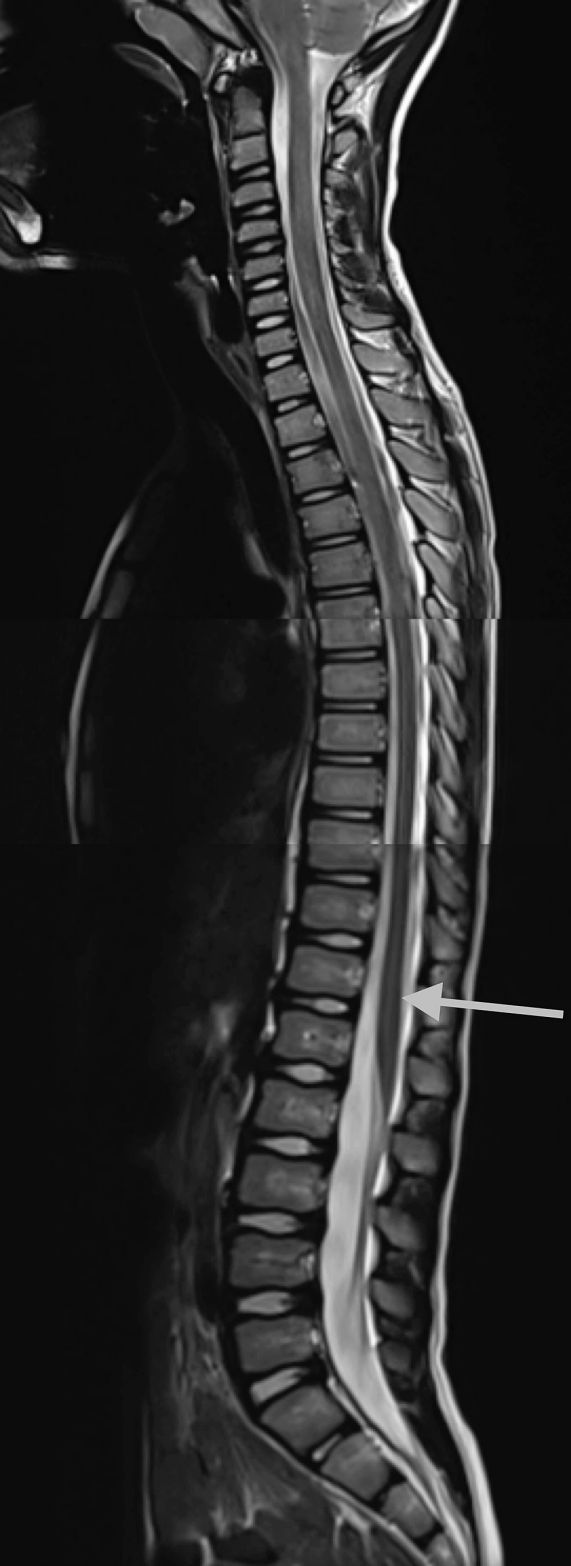
Stitched sagittal T2-weighted images of the cervical, thoracic, and lumbar spine at eight years of age show resolution of the slit-like syrinx, including in the lower cord (gray arrow)

**Figure 5:**
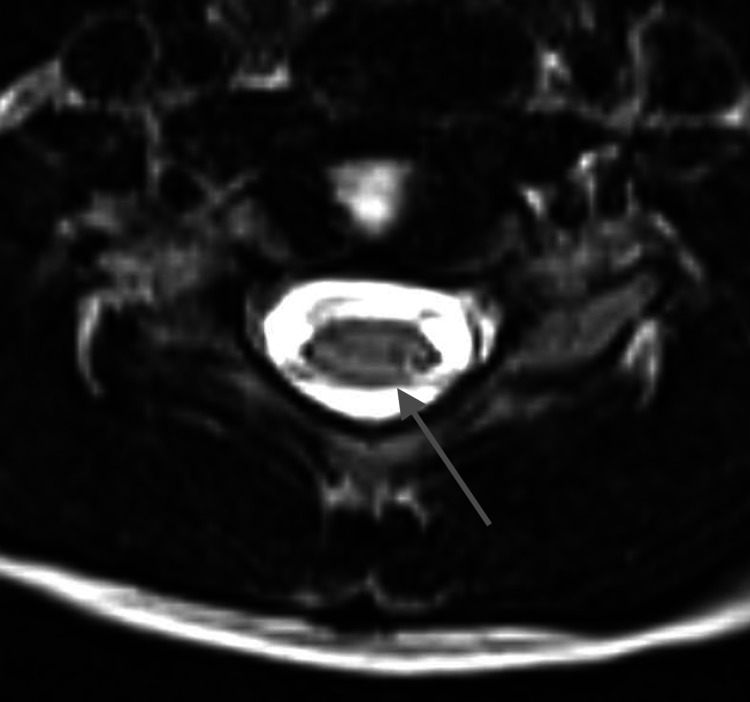
Axial T2-weighted image of the spine at the level of C6-7 at eight years of age shows resolution of the slit-like syrinx (gray arrow).

**Figure 6:**
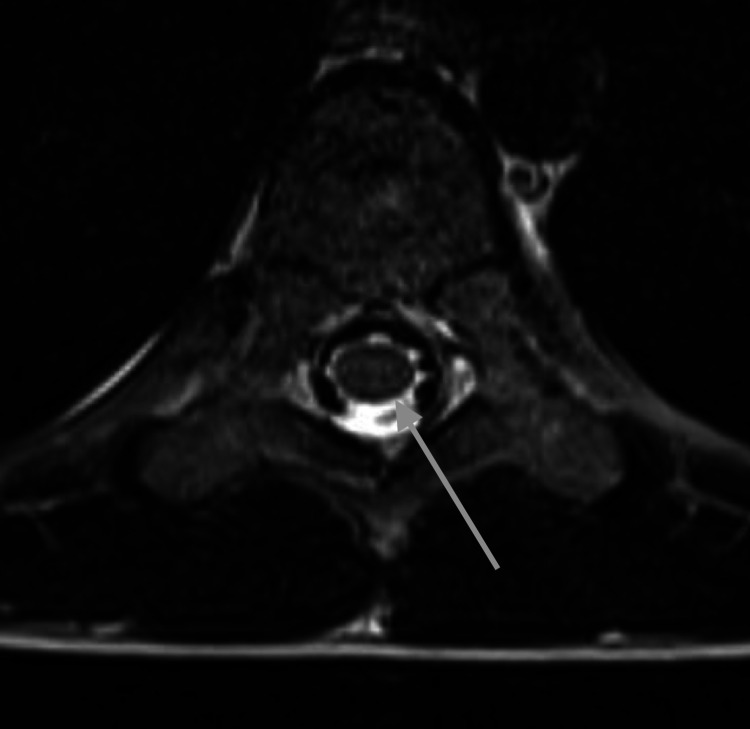
Representative axial T2-weighted image of the spine at the level of T9 at eight years of age shows resolution of the slit-like syrinx (gray arrow)

 A four-year-old male, born at full term, initially presented for brain magnetic resonance imaging (MRI) without contrast to evaluate for developmental delay, autism, and attention-deficit/hyperactivity disorder (ADHD). Images showed non-specific asymmetric prominence of the right lateral ventricle. There were no posterior fossa abnormalities. At the age of five years, the patient presented for a full spine MRI without contrast to evaluate for abnormal gait. T2-weighted images revealed a prominent central canal from the lower cervical cord and extending caudally (Figures 1-3). A few months later, at the age of six years, the patient was evaluated in the neurology clinic for autism spectrum disorder and possible syringomyelia. History revealed the patient had a tip-toe gait and a general lack of coordination. Other than gait abnormality, the neurologic examination was non-focal. The patient was diagnosed with syringomyelia, and a follow-up full spine MRI with and without contrast was performed six months later. The images from that time showed a similar prominent central canal measuring 0.5 mm in the lower cervical cord, 1 mm in the thoracic cord, and 2 mm in the distal cord, which ended at L1-2. There was no evidence of malignancy. On the same day as this imaging, the patient was evaluated in the neurosurgery clinic for thoracolumbar syringomyelia. No new symptoms were noted. The patient was diagnosed with holocord hydromyelia. Follow-up imaging was ordered, and a physical therapy consultation was arranged. A year and four months later, the patient was again evaluated in the neurosurgery clinic. The patient continued to have balance problems and difficulty in walking but had developed no new symptoms. The neurological exam was again non-focal, except for tip-toe gait. The patient was diagnosed with thoracolumbar syringomyelia. Follow-up imaging was ordered, and an orthotics consultation was arranged. Almost a year later, at eight years of age, the patient underwent a full spine MRI with and without contrast. Imaging findings were normal; specifically, the central canal was no longer prominent at any level (Figures 4-6).

## Discussion

The central canal is patent in utero with most levels usually occluded by early adult life [1-3]. An autopsy study of 232 patients revealed that some degree of central canal closure was present in no fetuses, 3% of infants, 18% of children, 88% of adolescents/young adults, 96% of middle-aged adults, and 100% of those 65 years and older [2]. The central canal ependymal lining changes from pseudostratified ciliated to simple columnar or simple cuboidal around the typical age of canal closure. There is a proliferation of ependyma and astrocytes, which break through the organized canal lining and continue to proliferate, ultimately occluding the central canal. It appears to be a disorganized process with astrogliosis and with the extent varying by level within an individual and in degree from person to person. Histologically, this process is more compatible with injury and scarring of the ependyma and less compatible with a degenerative or involutional process. This may account for the variation in the imaging presentation when the central canal is prominent; for example, holocord prominent central canals are more common in children, whereas focal prominent central canals are more common in adults [2]. In another histologic study, over 60% of patients in the one to five years cohort (n = 12) and over 60% of the patients in the six to nine years cohort (n = eight) had patent central canals at all levels from C3 to S3. All patients in the second-decade cohort (n = 12) had stenosis at some level and roughly 15% had stenosis at all levels [3].

The differential diagnosis for fluid signal in the distal spinal cord includes ventriculus terminalis (VT), which is a cystic, ependyma-lined cavity in the central conus medullaris. It represents a normal embryologic structure that usually undergoes regression in utero, though it can sometimes be seen in neonates [4-5] and rarely in adults [6]. Unlike the present case, however, VT is rounded and does not extend cranially above the conus medullaris [7].

There have been a few reported cases of spontaneous resolution or near resolution of the syrinx or prominent central canal in the absence of trauma and hindbrain malformations (Table 1). To our knowledge, we present the first case of an extensive/diffuse prominent central canal in a pediatric patient without a history of trauma or hindbrain abnormality that underwent spontaneous resolution.

**Table 1:** Characteristics of reported cases of spontaneous resolution of syrinx/prominent central canal in the absence of trauma and hindbrain malformations

Author	Age/Sex	Symptom(s)	Extent, Location	Symptomatic Course	Imaging Course	Follow-Up
Vinas [8]	58-year-old male	Right upper extremity paresthesias, neck pain	Focal, C6 to T1	Improved	Resolved	5 years
Kastrup [9]	61-year-old female	Chest wall paresthesias	Diffuse, C1 to conus	No change	Resolved	8 years
Ozisik [10]	30-year-old female	Right hemihypesthesia	Focal, C5 to C7	Resolved	Nearly resolved	6 years
Yeager [11]	19-month-old female	Seizure	Focal, C4 to C7	Resolved	Resolved	2 years
Present case	Five-year-old male	Abnormal gait	Diffuse, C6-7 to conus	No change	Resolved	3 years

Holly performed a retrospective analysis of 32 patients with focal slit-like syrinxes (mean diameter 2 mm, range 1-5 mm), who had long-term imaging follow-up (mean 38 months) and no hindbrain malformations [12]. All patients had some form of spinal symptoms but no to minimal findings on neurological exam. Over the follow-up course, six patients improved clinically, seven worsened, and the rest did not change. Interestingly, none of the syrinxes for any patient changed in size on follow-up imaging, making it unlikely that the reported symptoms were caused by the syrinxes. The slit-like syrinxes were defined by a consistently thin diameter, lack of associated symptoms, and lack of pathologic factors that alter the flow of cerebrospinal fluid (CSF). As these do not describe a pathologic process, in these cases, the authors advocated for using the term persistent central canal instead of *syringomyelia* to avoid diagnostic confusion and patient and physician anxiety.

## Conclusions

A persistent central canal is the result of delayed closure of the central canal of the spinal cord. We present a pediatric patient with a persistent central canal that underwent spontaneous normal closure, whose symptoms were unlikely to be related to the spinal imaging findings. Supporting the finding of a persistent central canal over a diagnosis of a syrinx, the canal diameter was consistently thin, and the patient did not have other findings to suggest altered CSF flow such as a hindbrain malformation. It is important to recognize that a prominent central canal can be a normal variant in order to avoid unnecessary anxiety, work-up, and intervention.
